# Advances in protein dot blot: principles, technical specifics, applications, and future perspectives

**DOI:** 10.3389/fmolb.2026.1768231

**Published:** 2026-01-23

**Authors:** Juan Lu, Feng-yi Mai, Xin-yu Li, Wen-tao Yang, Jing-rong Liang, Xing-long Li, Jie Guo, Chen-guang Li

**Affiliations:** 1 Shenzhen Hospital of Southern Medical University, Shenzhen, China; 2 Shenzhen Nanshan People’s Hospital, Affiliated Nanshan Hospital of Shenzhen University, Shenzhen, China; 3 Southern University of Science and Technology, Shenzhen, China; 4 Department of Breast and Thyroid Surgery, Union Hospital, Tongji Medical College, Huazhong University of Science and Technology, Wuhan, China; 5 Department of Rheumatology & Immunology, Shenzhen Second People’s Hospital, Shenzhen, China

**Keywords:** applications, high-throughput screening, protein detection, protein dot blot, technical specifics

## Abstract

Protein dot blot is an efficient immunoblotting technique enabling qualitative/semi-quantitative protein analysis without electrophoresis, relying on antigen-antibody binding. Its workflow involves direct sample spotting on membranes, blocking, antibody incubation, and signal detection, completing within 3–5 h. Advantages include simplicity, high throughput, micro-sample compatibility, and cost-effectiveness, supporting basic life science research and clinical testing. However, it faces limitations like narrow dynamic range, inability to resolve protein variants, susceptibility to non-specific binding, and sensitivity to operational variables. This review systematically elaborates on its principles and procedures, analyzes key factors influencing sensitivity, and repeatability, and focuses on recent application progress in protein analysis, clinical biomarker detection, and food safety, along with technical innovations. It aims to provide comprehensive references for researchers and a theoretical basis for further optimization, with future advancements likely involving nanomaterial-based signal amplification, engineered antibodies, and integration with microfluidics or mass spectrometry to expand utility in biomedicine and public health.

## 1 Introduction

Proteins are pivotal executors of biological processes, and their expression profiling is essential for deciphering disease mechanisms and identifying biomarkers or therapeutic targets (Towbin et al., 1979; Kurien and Scofield, 2015b). Accurate, high-throughput protein detection is critical for unraveling physiological/pathological roles, yet traditional antibody-based methods (e.g., Western blot and enzyme-linked immunosorbent assay (ELISA)) have inherent limitations. The Western blot assay serves as an initial tool in laboratory research for identifying proteins of interest. However, this technique is time-consuming, allows only a limited number of samples to be assayed simultaneously, and entails multiple procedural steps. Consequently, it is often impractical to process all samples collected from a study design in a single experimental run, especially when ELISA, on the other hand, offers quantitative advantages and higher throughput via microplate formats but relies on solid-phase adsorption in wells, which may introduce variability in protein binding efficiency (Simpson et al., 2009). For this reason, there is a need for novel and innovative methodologies enabling high-throughput protein identification and quantification. Such approaches would mitigate the inherent variability introduced by conducting experiments across multiple sessions, which is particularly crucial when researchers aim to analyze complex signal transduction pathways involving various proteins (e.g., enzymes, receptors) or metabolites associated with a specific signaling cascade.

Protein dot blot assay has emerged as an alternative strategy that eliminates the need for protein separation while reducing the required amounts of sample and antibodies. This techniques shares similarities with ELISA; however, instead of utilizing standard well plates, they employ nitrocellulose (NC) or polyvinylidene fluoride (PVDF) membranes for protein immobilization and subsequent execution of the classic immunodetection procedure, thereby enabling quantitative detection of the target protein (Towbin et al., 1979; Simpson et al., 2009; Tan et al., 2011). The protein dot blot assay exhibits distinct experimental advantages that render it a valuable tool in molecular biology research. Notably, it enables rapid and high-throughput analysis of multiple samples, as the procedure circumvents the need for labor-intensive protein separation steps typically required in techniques such as SDS-PAGE. This not only reduces assay time but also minimizes the consumption of precious samples and reagents, including antibodies, an attribute particularly beneficial when working with limited biological materials. Furthermore, the method offers robust quantitative capabilities, with signal intensities correlating well with target protein concentrations, thereby facilitating accurate relative or absolute quantification. Its simplicity in operation and compatibility with various detection systems further enhance its versatility, making it adaptable to diverse experimental settings ranging from basic research to clinical diagnostics. Collectively, these advantages underscore the protein dot blot as a cost-effective, efficient, and reliable approach for protein analysis. Thus, the protein dot blot has carved a unique niche in molecular biology by addressing critical limitations of traditional methodologies. This review comprehensively summarizes the protein dot blot assay, covering its fundamental principles, standard protocols, critical technical considerations, and diverse applications. It aims to fill existing literature gaps by integrating technical essentials with recent innovations, providing a clear methodological reference for researchers and highlighting optimizations that expand the technique’s utility. Ultimately, this review underscores the assay’s unique niche in molecular biology and its potential to advance protein analysis workflows.

## 2 Principle of the protein dot blot technique

Protein dot blot is an immunoblotting assay for qualitative detection and semiquantitative analysis of a target proteins (Stott, 1989). Fundamentally, it shares the same core principle as Western blot: the specific binding between an antigen (the target protein) and an antibody, which serves as the molecular basis for selectively recognizing and capturing the protein of interest from complex biological samples (Hawkes et al., 1982). Unlike Western blot, which requires electrophoretic separation of proteins by size followed by transfer of protein bands from a gel to a membrane, dot blot bypasses these steps entirely. Instead, the sample containing the target protein is directly spotted onto a solid membrane, typically NC or PVDF membrane (Goldman et al., 2016). These membranes have high affinity for proteins, allowing them to irreversibly bind to the membrane surface through hydrophobic interactions and electrostatic forces, immobilizing the protein at the spot where the sample was applied. Following immobilization, the membrane undergoes standard immuno-detection steps to visualize the target protein. The membrane is first blocked to minimize non-specific antibody binding and background noise, then incubated with a primary antibody specific to the target protein. After washing to remove unbound primary antibodies, a labeled secondary antibody (conjugated to enzymes like HRP or fluorescent dyes) is added to form a detectable immune complex. Finally, a substrate triggers a signal-producing reaction (catalyzed by the enzyme or via fluorescence), with signal presence indicating target protein existence and signal intensity reflecting relative abundance—enabling semiquantitative analysis through densitometry or visual assessment (Stott, 1989; Ghorbanizamani et al., 2021). This direct and streamlined workflow facilitates rapid readout, with results achievable significantly faster than those from conventional Western blot, while enabling high-throughput screening by allowing parallel analysis of dozens or even hundreds of samples. Thus, the protein dot blot technique leverages antigen-antibody specificity, solid-phase immobilization, and signal amplification via labeled conjugates to efficiently detect specific proteins, making it a valuable tool in research, diagnostics, and protein analysis, particularly in scenarios requiring quick confirmation of protein expression or high-throughput sample screening.

## 3 Experimental procedures for protein dot blot analysis

### 3.1 Sample preparation

Proteins were extracted from appropriate sources based on experimental requirements. For instance, tissues or cell pellets were lysed (typically using lysis buffer with optional sonication) to obtain total protein extracts; culture supernatants, serum, urine, or other fluid samples were used directly or after appropriate dilution. To prevent proteolysis, protease inhibitors (e.g., phenylmethylsulfonyl fluoride, PMSF) were included during extraction. Protein concentrations were determined using assays such as BCA or Lowry to ensure samples were adjusted to optimal concentrations for spotting (Helbing et al., 2022).

### 3.2 Blotting membrane selection and preparation

A suitable membrane type, either NC or PVDF membrane was selected and cut to the desired dimensions. For PVDF membranes, activation was performed by soaking in absolute methanol for a suitable duration until uniformly translucent in appearance, followed by brief rinse with deionized water and equilibration in buffer. NC membranes did not require methanol activation and were directly wetted with deionized water or buffer. A corner of the membrane was marked (e.g., via a notch in the top-left corner) to indicate orientation, facilitating subsequent spot localization. The membrane was then laid flat on a clean, solid surface (e.g., glass or plastic plate) with bubble-free contact ensured (Xiang et al., 2021).

### 3.3 Sample loading and fixation

Prepared protein sample was applied as discrete spots, with appropriate controls included to validate assay specificity and quantification reliability—specifically, positive controls (known target protein or verified positive samples) and negative controls (protein-free samples) (Kurien and Scofield, 2015a). After spotting, the membrane was air-dried at room temperature or dried in an incubator, taking care to avoid overheating-induced protein denaturation or membrane damage. Drying enhanced stable adsorption of proteins to the membrane via hydrophobic and electrostatic interactions, preventing elution during subsequent steps and ensuring signal stability (Helbing et al., 2022).

### 3.4 Blocking and antibody incubation

Non-specific binding sites on the membrane are blocked using a suitable blocking solution, with common choices including nonfat dry milk or bovine serum albumin (BSA) dissolved in PBST buffer; this step is critical for minimizing background noise (Goldman et al., 2016). Following removal of the blocking solution, primary antibody incubation is conducted with antibodies specific to the target protein, followed by washing to remove unbound primary antibody. Secondary antibody conjugated to an enzymes (e.g., horseradish peroxidase) or fluorophores are then applied, with species compatibility ensuring specific binding to the primary antibody. Additional washes post-secondary antibody incubation further reduce non-specific signals (Ghorbanizamani et al., 2021; Greenfield, 2021).

### 3.5 Signal detection

Detection strategies are tailored to the secondary antibody label. Enzyme-conjugated secondary antibodies utilize chemiluminescent substrates, with signal capture via imaging systems or X-ray film. Fluorescently labeled secondary antibodies are visualized using fluorescence imaging devices, enabling direct spot signal quantification (Ghorbanizamani et al., 2021).

**FIGURE 1:**
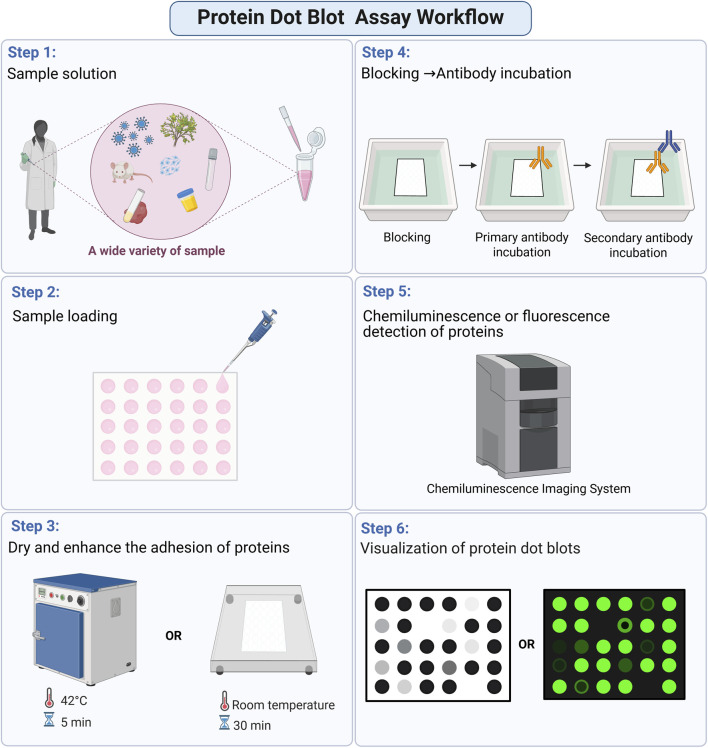
Schematic ilustration of the protein dot blot detection workflow.

The core workflow of protein dot blot comprises sequential key stages: sample preparation, blotting membrane preparation, sample loading, blocking, antibody incubation, and signal detection (Figure 1). Unlike traditional Western blot, this technique eliminates intermediate steps such as gel electrophoresis and membrane transfer, enabling streamlined sample processing and detection. These efficiencies make protein dot blot an optimal choice for high-throughput screening of large sample cohorts.

## 4 Key technical points and precautions for protein dot blot

### 4.1 Dot blot membrane selection

Two commonly used membranes in biochemical analyses are NC and PVDF membranes (Xiang et al., 2021). NC membranes, characterized by their cost-effectiveness, possess a porous, mesh-like structure with typical pore sizes of 0.45 µm or 0.22 µm. They exhibit a protein-binding capacity ranging approximately from 80 to 150 μg/cm^2^ and can be readily utilized upon wetting with an appropriate buffer. Nevertheless, a notable limitation of NC membranes lies in their relative brittleness, rendering them susceptible to tearing during handling. In contrast, PVDF membranes demonstrate superior mechanical robustness, including enhanced tear resistance, chemical stability, and high mechanical strength. Moreover, they offer a higher protein-binding capacity, generally in the range of 100–200 μg/cm^2^. PVDF membranes are particularly advantageous for applications involving low-abundance proteins or samples containing detergents, as they tend to retain proteins with greater affinity (Goldman et al., 2016; Xiang et al., 2021). The selection of membrane type is inherently dependent on the specific experimental context. For high-abundance target proteins (e.g., housekeeping proteins such as β-actin or GAPDH) when ample sample material is available, NC membranes are often sufficient and can generate strong signals (Xiang et al., 2021). Conversely, for low-abundance targets (e.g., certain signaling proteins or transcription factors) or when sample quantities are limited, PVDF membranes are generally preferred due to their ability to achieve higher capture efficiency and, consequently, stronger signal intensity. Thus, the appropriate choice of membrane material is crucial to ensuring optimal retention and detectability of the target protein.

### 4.2 Sample concentration and spot volume

Both the protein concentration in the sample and the volume of the spotted aliquot represent critical parameters requiring systematic optimization. Fine-tuning these two factors not only directly influences the uniformity of spot morphology but also dictates whether the signal intensity exhibits a linear correlation with protein concentration. When protein concentrations exceed the binding capacity of the membrane, the available binding sites on the membrane surface become rapidly saturated; surplus proteins, unable to form stable interactions, diffuse outward alongside the spotting solution. This diffusion leads to the formation of enlarged spots with indistinct, blurred edges. In extreme cases, continued diffusion can result in overlap between adjacent spots, thereby inducing signal cross-contamination and complicating the discrimination of individual samples. Conversely, when the protein concentration falls below the membrane’s effective capture threshold, the molecular density within the spot becomes insufficient to support efficient binding. Under such circumstances, increasing the spotting volume fails to enhance detectability, as the resulting signal remains either absent or indistinguishable from background noise, rendering it difficult to differentiate from negative controls. For low-abundance proteins, excessively dilute samples further exacerbate experimental variability and diminish the reproducibility of replicate assays. To mitigate these challenges, the implementation of a preliminary titration experiment is imperative. By evaluating a range of sample dilutions and spotting volumes, researchers can establish optimal conditions wherein the signal intensity of the spots displays a linear relationship with protein concentration across a defined range (Haab et al., 2001).

### 4.3 Selection of blocking solutions

Common blocking reagents include non-fat milk and bovine serum albumin. Non-fat dry milk, typically used at a 5% concentration in PBS with Tween-20 (PBST), is cost-effective and generally exhibits good blocking efficacy (Mishra, 2022). However, its casein component can interfere with the detection of phosphorylated proteins: casein, being a phosphorylated protein itself, may non-specifically bind to anti-phospho antibodies, thereby elevating background signals. In such scenarios, BSA commonly used at 3%–5% in PBST is the preferred alternative, as it lacks phosphorylation sites and thus minimizes cross-reactivity (Kee et al., 2013). Ultimately, the selection of an appropriate blocking buffer should be determined based on its compatibility with both the antibody and the target protein of interest.

### 4.4 Antibody dilution ratio and washing optimization

Optimal dilution of primary and secondary antibodies balances specific signal intensity and background noise (Goldman et al., 2016). Over-dilution (insufficient antibody concentration) leads to suboptimal antigen binding, resulting in weak signals or false negatives. Conversely, excessive antibody concentration promotes non-specific binding to irrelevant sample components, increasing background interference and obscuring true signals. To determine the optimal dilution, a gradient of antibody concentrations should be systematically evaluated. The ideal dilution is defined as the condition that yields a distinct, target-specific signal with negligible background. Rigorous optimization of antibody dilution ratios is therefore indispensable for ensuring the reproducibility and accuracy of dot blot assays.

### 4.5 Washing conditions

Thorough washing between each incubation is essential for reducing background and improving the signal-to-noise ratio. Inadequate washing may result in residual unbound or weakly associated antibodies, which can contribute to non-specific signal generation. A recommended protocol involves washing the membrane a minimum of three times following each antibody incubation, with each wash lasting approximately 5 min (Stott, 1989). These washes should utilize a sufficient volume of washing buffer to ensure complete immersion of the membrane, accompanied by gentle agitation to facilitate the removal of loosely bound components. Beyond the aforementioned key factors, numerous procedural nuances can impact the consistency of dot blot results. For instance, the hydration state of the membrane during spotting is critical: uneven drying of membrane regions during spotting can lead to erratic behavior of applied sample droplets (Greenfield, 2021). Such irregularities may manifest as “donut-shaped” spots, characterized by concentrated signal at the edges and a diminished center, arising from partial absorption followed by abrupt cessation of droplet penetration. To mitigate this, the membrane must be maintained in a uniformly moist state throughout the spotting process, with operations performed expeditiously to prevent evaporation of microdroplets prior to complete absorption. Additionally, the spotting technique itself specifically the steadiness and speed of application, influences spot morphology; excessively slow spotting can promote undue spreading or uneven drying of the sample. Incubation parameters, including temperature and gentle agitation, must be standardized across experiments to ensure reproducibility.

In summary, the dot blot technique is highly dependent on strict protocol standardization (Table 1). Minor deviations in handling can lead to disproportionately large variations in results. To obtain reliable data, researchers must adhere closely to optimized procedures and maintain consistent conditions throughout all stages, including membrane preparation, spotting, blocking, antibody incubation, and washing. Meticulous attention to these details forms the foundation for achieving high repeatability and robust confidence in dot blot outcomes.

**TABLE 1:** Key technical considerations and critical precautions for Dot Blot Analysis.

Technical aspect	Key details and rationale	Critical precautions
Membrane selection	NC membrane: Cost-effective, pore size 0.45/0.22 µm, protein-binding capacity 80–150 μg/cm^2^, ready for use after buffer wetting	Choose NC membrane for high-abundance targets (e.g., β-actin, GAPDH) with sufficient sample
	PVDF membrane: Superior mechanical strength/chemical stability, binding capacity 100–200 μg/cm^2^, high affinity for proteins	Strong; proteins are resolved by SDS-PAGE according to molecular weight
Sample concentration & spot volume	Both parameters determine spot uniformity and linearity between signal intensity and protein concentration. Optimal conditions require a linear signal-concentration relationship within a defined range	Avoid over-concentration (exceeds membrane binding capacity): Causes spot diffusion, blurred edges, or adjacent spot overlap (cross-contamination)
		Avoid under-concentration (below capture threshold): Results in undetectable signals or background-like noise
		Perform preliminary titration of dilutions/volumes for low-abundance proteins to ensure reproducibility
Blocking solution selection	Non-fat dry milk (5% in PBST): Cost-effective, good general blocking efficacy	Avoid non-fat milk for phosphorylated protein detection (casein binds anti-phospho antibodies, increasing background)
	BSA (3%–5% in PBST): Phosphorylation-site free, minimizes cross-reactivity	Select based on compatibility with primary antibody and target protein
Antibody dilution ratio	Primary and secondary antibodies require optimization to maximize specific signal and minimize background	Avoid over-dilution (insufficient binding, weak signals/false negatives)
		Avoid under-dilution (excessive non-specific binding, high background obscuring true signals)
		Test a concentration gradient to identify conditions with distinct target signals and negligible background
Washing conditions	Post-incubation washes reduce unbound antibody residues; recommended protocol: ≥3 washes (5 min each) with sufficient PBST, gentle agitation	Ensure complete membrane immersion to avoid uneven washing
		Maintain consistent agitation to remove loosely bound components
		Inadequate washing leads to non-specific signal generation
Procedural nuances	Membrane hydration: uniform moisture ensures consistent sample absorption	Prevent membrane drying during spotting (causes “donut-shaped” spots with edge-concentrated signals)
	Spotting technique: steady, rapid application prevents spreading/uneven drying	Perform spotting expeditiously to avoid microdroplet evaporation
	Incubation parameters: standardized temperature and gentle agitation	Standardize all incubation conditions across experiments for reproducibility

## 5 Advantages and limitations of protein dot blot

### 5.1 Advantages

#### 5.1.1 Broad sample applicability

The dot blot technique exhibits remarkable versatility in accommodating diverse sample types and sources. It is compatible with complex biological mixtures, including cell or tissue lysates, serum, plasma, urine, cerebrospinal fluid, cell culture supernatants, and other biological fluids or extracts. Unlike Western blot, which mandates relatively purified and solubilized protein samples, dot blot tolerates variations in protein solubility and sample purity with minimal preprocessing, enabling analysis of recalcitrant or low-abundance proteins. In contrast, ELISA is prone to protein loss and denaturation due to solid-phase adsorption, limiting its applicability to samples with sufficient protein yield (Norouzi et al., 2025). This broad adaptability significantly expands the range of experimental scenarios in which the dot blot technique can be effectively applied.

#### 5.1.2 Operational efficiency: simplicity, rapidity, and cost-effectiveness

Dot blot exhibits remarkable operational efficiency by integrating simplicity, rapid turnaround, and cost-effectiveness. Unlike Western blot, which requires time-consuming electrophoretic separation and transfer steps (often taking several days to complete), dot blot streamlines the workflow by eliminating these procedures, enabling assay completion within hours. This efficiency makes it ideal for high-throughput screening and time-sensitive analyses (Mishra, 2022). Additionally, dot blot obviates the need for expensive electrophoresis equipment and associated reagents, resulting in significantly lower overall costs compared to Western blot. While ELISA also offers high throughput, dot blot outperforms it in terms of operational simplicity and cost-efficiency, as ELISA involves more complex solid-phase adsorption processes and higher reagent consumption. The simplified protocol also reduces technical barriers, facilitating adoption by novice users and ensuring consistent results across parallel analyses of tens to hundreds of samples on a single membrane. In terms of analytical utility, dot blot enables semi-quantitative assessment of target proteins by comparing spot signal intensities, offering a more direct and efficient readout unencumbered by variations in electrophoretic mobility. Qualitative results can be obtained by simply noting the presence or absence of a spot, which indicates whether the target protein is present in a sample. Moreover, quantitative comparison of relative protein abundance across samples can be performed using image analysis software to measure spot optical density, thereby enhancing the objectivity and precision of the results (Kurien and Scofield, 2015a; Goldman et al., 2016).

#### 5.1.3 Minimal sample requirement

Dot blot assays require only microliter-scale volumes or nanogram quantities of protein per spot, a feature that confers substantial advantages when working with limited biological material, such as cell lysates, biopsy-derived tissues, or rare clinical specimens. This minimal input requirement not only preserves precious samples for replicate analyses or complementary experimental approaches but also facilitates parallel evaluation of multiple specimens on a single membrane. In contrast, Western blot requires 10–30 μg of protein per lane and relies on electrophoretic separation, failing to meet the needs of micro-samples like single-cell lysates (Bass et al., 2017). Although ELISA offers high throughput, it necessitates 50–100 μL of sample per well and nanogram to microgram-scale protein; moreover, solid-phase adsorption often causes protein loss, requiring higher sample input for compensation (Tabatabaei and Ahmed, 2022). By enabling direct membrane immobilization without separation, dot blot preserves precious samples for replicate assays or complementary analyses while facilitating parallel detection of multiple samples. By virtue of these attributes, dot blot technology serves as a pragmatic platform for high-throughput preliminary screening, enabling efficient detection of relative differences between samples and thereby guiding subsequent in-depth investigations (Galperin et al., 2004; Mishra, 2022).

#### 5.1.4 High detectability

Exhibiting high detectability, dot blot assays can achieve picogram-level sensitivity when coupled with high-affinity antibodies, a performance comparable to that of Western blot (Bass et al., 2017; Macala et al., 2024). Notably, it detects as low as 0.19 ± 0.04 pmol of protein carbonyls using merely ∼60 ng of total protein (Wehr and Levine, 2012), whereas Western blot requires 10–30 μg per lane (100–1000-fold higher input) (Bass et al., 2017), rendering it unsuitable for scarce samples like single-cell lysates or biopsies—an application where dot blot emerges as irreplaceable. In contrast to ELISA, whose sensitivity fluctuates from picogram to nanogram levels and is compromised by protein loss/denaturation from solid-phase adsorption (Tabatabaei and Ahmed, 2022), dot blot enables direct immobilization on NC/PVDF membranes, ensuring superior practical detectability in complex matrices. For example, gold nanoparticle-amplified dot blot detects Alzheimer’s-related Aβ_1-42_ at 50 pg/mL in CSF, outperforming commercial ELISA kits (100–200 pg/mL) (Wang et al., 2012). Further enhanced by signal amplification technologies, dot blot uniquely integrates high sensitivity with minimal sample consumption, ideal for low-abundance biomarker screening and rare protein analysis. Owing to its time efficiency, operational simplicity, high-throughput capability, minimal sample consumption, and cost-effectiveness, the dot blot technique occupies a prominent position in protein analysis. It is particularly valuable for preliminary screening of target proteins, comparative evaluation of large sample cohorts, and rapid validation of experimental results.

### 5.2 Limitations

Despite its strengths in rapid qualitative and semi-quantitative analysis, dot blot has inherent limitations stemming from its simplified workflow and operational principles. The relationship between spot intensity and protein concentration is linear only within a restricted range (Macala et al., 2024). At high protein levels, spots may saturate, failing to reflect proportional concentration increments, while near the detection threshold, signals may become indistinguishable from background noise. This narrow dynamic range compromises the technique’s reliability for precise quantification. In addition, because proteins are directly applied to the membrane without electrophoretic separation, the method cannot separate proteins of similar molecular weight, different isoforms, or post-translational modifications (Mishra, 2022). For instance, phosphorylated versus unphosphorylated variants, full-length proteins versus cleavage products, or active versus inactive conformers will yield merged signals, obscuring molecular heterogeneity. Another critical limitation is the risk of non-specific binding. Since dot blot relies exclusively on antibody specificity, cross-reactivity with irrelevant proteins in complex matrices (e.g., serum or tissue homogenates containing thousands of proteins) can generate false-positive signals, complicating result interpretation. The technique is also highly sensitive to experimental handling variables. Fluctuations in spotting volume, pipetting speed, membrane hydration status, or washing efficiency can profoundly alter spot morphology, impeding reproducibility across experimental runs or laboratories. If loading is too slow may cause partial droplet evaporation prior to membrane absorption, resulting in “hollow spots” with intensified edges and attenuated centers (Galperin et al., 2004). Similarly, uneven membrane wetting can induce irregular protein diffusion, leading to inconsistent spot shapes (Carpenter et al., 2008). Over-washing may remove target proteins that are only loosely bound to the membrane, while insufficient washing may elevate background noise. Additionally, large protein complexes may bind inefficiently to the membrane matrix, and highly hydrophobic proteins may aggregate or precipitate during spotting (Pillai-Kastoori et al., 2020).

Notably, the lack of electrophoretic separation precludes the use of electrophoretic mobility to infer molecular weight, isoelectric point, or other physicochemical properties of the target protein. It also provides no insights into structural states such as cleavage, oligomerization (e.g., dimer or multimer formation), or conformational changes. Thus, for studies requiring detailed protein characterization, dot blot cannot substitute for Western blot or other high-resolution methods. Instead, its utility is best confined to rapid qualitative screening and semi-quantitative comparisons, with applications demanding precise quantification, complex sample analysis, or mechanistic investigations necessitating complementary approaches such as ELISA, Western blot, or mass spectrometry.

## 6 Application of protein dot blot

### 6.1 Basic research applications

#### 6.1.1 Recombinant protein analysis

Protein dot blot stands as one of the most prevalent and invaluable techniques in the rapid screening of recombinant protein expression (Singh and Baldock, 2020; Mishra, 2022; Guo et al., 2025). Conventionally, verifying the expression of a cloned gene product, such as in *Escherichia coli* or cultured cells, often entails running multiple SDS-PAGE gels followed by Western blot (Abolliel and Zedan, 2015). Protein dot blot is particularly advantageous in high-throughput screening, where multiple clones or culture conditions, such as induction time, temperature, and inducer concentration, can be compared side by side on a single membrane. Positive clones or favorable conditions yield strong immunoreactive dots, while negative ones show no signal, allowing immediate visual discrimination (Mishra, 2022). Morris et al. (2023) demonstrated an automated platform that monitors bacterial growth and total protein content in well plates, while simultaneously using dot blot assays to specifically quantify the target protein of interest. This approach facilitated the rapid identification of high-yield expression conditions within 1 week and even rescued previously non-expressible proteins by systematically exploring expression parameters. These results collectively illustrate the potential of dot blot to significantly shorten the experimental cycle, providing a rapid and reliable foundation for optimizing protein expression workflows. As an efficient preliminary tool, it offers researchers quick and reliable results in the early stages of recombinant protein studies. Moreover, the application of dot blot is not limited to conventional recombinant proteins; it can also be effectively used for detecting secreted and membrane proteins (Singh and Baldock, 2020; Mishra, 2022). Additionally, this technique is suitable for evaluating the effects of variables such as viral multiplicity of infection and infection duration on protein expression.

#### 6.1.2 Protein modification and conformational analysis

For the detection of protein modifications (e.g., carbonylation, a key oxidative stress biomarker), dot blot offers superior sensitivity and minimal sample requirement compared to Western blot. Unlike Western blot, which demands microgram-scale protein input and limits high-throughput applications, dot blot achieves picomolar-level detection with only nanogram quantities of protein, enabling analysis of scarce samples (e.g., single-organism extracts) and large sample cohorts (Wehr and Levine, 2012; Arur and Schedl, 2014; Cao et al., 2025; Guo et al., 2025; Wei et al., 2025). The measurement of protein carbonyls can be achieved through derivatization with 2,4-dinitrophenylhydrazine (DNPH), followed by probing with anti-carbonyl antibodies. Within this assay system, dimethyl sulfoxide is employed as the solvent due to its ability to efficiently extract proteins from tissues while preserving their solubility. Under these optimized conditions, the detection limit can reach 0.19 ± 0.04 pmol of carbonyls, with only approximately 60 ng of protein required per spot. Such high sensitivity enables the detection of oxidative damage even in samples extracted from a single fruit fly, and multiple samples can be concurrently analyzed on a single PVDF membrane. Beyond qualitative identification, the intensity of the spots allows for semi-quantitative comparison of modification levels across different samples, which furnishes valuable insights for investigating oxidative stress-related physiological and pathological processes (Wehr and Levine, 2012; Arur and Schedl, 2014). In viral protein research, dot blot facilitates the investigation of conformational dynamics by leveraging epitope-specific antibodies to monitor structural rearrangements under physiological or experimental conditions. This approach avoids the complexity of high-resolution structural techniques while providing rapid, direct readouts, supporting mechanistic studies on viral entry and antiviral drug development (Sari et al., 2020).

#### 6.1.3 Exosome protein analysis

Exosomes, nanoscale vesicles enclosed by a phospholipid bilayer, are ubiquitously distributed in biological fluids such as plasma and urine. Shielded by their lipid membrane, exosomal proteins exhibit enhanced stability in their post-translationally modified forms, thereby offering a more precise reflection of physiological and pathological states (Momenbeitollahi et al., 2022; Dilsiz, 2024; Wantchecon et al., 2025). This unique characteristic renders exosomes an ideal source of disease biomarkers. However, the relatively low abundance of exosomes in complex biological matrices, coupled with the inefficiencies of traditional isolation techniques like ultra-centrifugation, has impeded their widespread application in large-scale clinical investigations. To tackle these challenges, Hu and colleagues (Feng et al., 2025) recently developed an amphiphilic supramolecular probe, which enables highly efficient capture of exosomes from minimal volumes of clinical samples. By coating this probe onto NC membranes, exosomes can be immobilized in an array format, a design that not only enhances throughput but also circumvents the protein loss and denaturation commonly encountered in electrophoretic transfer steps. In a clinical validation study, the research team analyzed plasma samples from 36 hepatocellular carcinoma patients and 12 healthy controls, quantifying PD-L1 (a well-recognized immunotherapy biomarker) and Syntenin-1 (a canonical exosome marker). Notably, only 1 µL of plasma was required per sample, and the assay demonstrated high sensitivity with excellent quantitative accuracy (Feng et al., 2023). These findings highlight the potential of the dot blot-based exosome array as a robust solution for high-throughput liquid biopsy, providing a scalable strategy for biomarker screening in large clinical cohorts.

#### 6.1.4 Bacterial protein detection

The dot blot format exhibits high adaptability for pathogen detection assays, facilitating the identification of either microbial antigens or pathogen-specific antibodies in clinical samples. Its rapidity and operational simplicity render it particularly valuable for infectious disease diagnosis and epidemiological screening, especially in resource-constrained settings or high-throughput scenarios. Over the years, dot blot has been developed for a broad spectrum of pathogens, including bacteria, viruses, and parasites, offering the advantages of rapid result turnaround and multiplex target detection capabilities (Correa et al., 2007; Cho et al., 2024; Nguyen et al., 2025; Haugsten et al., 2026; Miao et al., 2026). A paradigmatic application lies in the detection of diverse serotypes of *Salmonella enterica*, a major bacterial pathogen responsible for foodborne illnesses (Crum-Cianflone, 2008). With over 2,600 serotypes, *Salmonella* is classified based on distinct surface antigens. Conventional culture-based serotyping methods are notoriously time-intensive, whereas dot blot assays have emerged as a streamlined alternative. This approach involves immobilizing either antigen extracts or serotype-specific antibodies onto a membrane, followed by probing with the corresponding target molecules from test samples. For instance, antibodies specific to *Salmonella* O:9 (serotype *Enteritidis*) and O:4 (serotype *Typhimurium*) can be co-immobilized on a membrane, enabling simultaneous multiplex detection of both serotypes in a single reaction. In spiked egg samples, such a dot blot assay demonstrated >90% specificity, effectively differentiating *S. Enteritidis* from other serotypes and non-Salmonella bacteria (e.g., *E.coli*). Field trials conducted at poultry slaughterhouses revealed approximately 85% concordance with traditional culture methods, while reducing the detection time from several days to merely 3–4 h (Yoshimasu and Zawistowski, 2001). These findings highlight the significant utility of dot blot assays in food safety surveillance, outbreak investigations, and rapid serotype-specific pathogen detection.

#### 6.1.5 Parasitic infection detection

Parasitic infections represent another filed where dot blot has demonstrated substantial utility. In the case of *Toxoplasma gondii*, the causative agent of toxoplasmosis, diagnosis often relies on detecting stage-specific antibodies. A key diagnostic marker is the dense granule antigen GRA8, which is robustly expressed during the early stages of host cell invasion but undergoes downregulation in chronic infections (Costa and Dure, 2016; Costa and Vilarino, 2018; Safarpour et al., 2021). The detection of anti-GRA8 antibodies in serum via dot blot assays thus enables differentiation between acute and chronic toxoplasmosis, facilitating timely intervention and mitigating the risks of maternal-fetal transmission and neurological sequelae. Furthermore, safarpour and colleagues developed a nanoparticle-based dot blot assay capable of simultaneously detecting anti-*Toxoplasma* IgM and IgG in patient specimens (Costa and Dure, 2016; Costa and Vilarino, 2018; Safarpour et al., 2021). Using a sandwich format integrated with chitosan-gold nanoparticle conjugates, this assay generates visually discernible dots and maintains high sensitivity even at low antibody titers. Such advancements enhance the value of dot blot technology for point-of-care testing in resource-constrained environments, as well as for the clinical management of toxoplasmosis in pregnant women and immunocompromised individuals.

### 6.2 Clinical samples analysis

#### 6.2.1 Neurodegenerative disease biomarkers

Dot blot has emerged as a promising tool for detecting low-abundance neurodegenerative disease biomarkers (e.g., Aβ peptides in Alzheimer’s disease) (Permanne et al., 1995; Bhuiya et al., 2025; Perbet et al., 2025). Wang and colleagues (Wang et al., 2012) developed a gold nanoparticle amplified dot blot immunoassay for Aβ_1-42_. In this approach, antibodies conjugated to gold nanoparticles serve to amplify the signal on the membrane, yielding a detection limit as low as 50 pg/mL. This level of sensitivity is comparable to, or exceeds that of, many ELISAs. Notably, the method was validated using cerebrospinal fluid (CSF) samples derived from AD patients. Given the well-established correlation between CSF Aβ concentrations and disease status, the capacity to detect sub-picogram amounts of Aβ paves the way for earlier diagnosis of AD and monitoring of therapeutic responses.

#### 6.2.2 Autoantibody screening in autoimmune disorders

Dot blot offers a pragmatic strategy for validating candidate autoantigens in autoimmune disorders. In systemic lupus erythematosus (SLE), diagnosis has conventionally depended on the detection of multiple autoantibodies, including anti-nuclear antibodies (ANA), anti-double-stranded DNA, and anti-Sm antibodies (Prasad et al., 1989; Kurien and Scofield, 2006). To explore novel biomarkers, autoantibodies targeting TCP1 were investigated using dot blot analysis. Recombinant TCP1 protein, expressed in SF9 cells, was spotted onto membranes, and sera from SLE patients, individuals with other autoimmune diseases (encompassing rheumatoid arthritis, systemic sclerosis, and Behçet’s disease), and healthy controls were applied. The dot blot results revealed that anti-TCP1 antibodies were present in 79% of SLE patients, while being rarely detected in the other groups. This corresponded to a diagnostic sensitivity of 79% and a specificity of 90%. These findings illustrate that dot blot can be effectively utilized to validate TCP1 autoantibodies as SLE-specific biomarkers (Nezlin, 2000; Lee et al., 2024). In summary, this method facilitates high-throughput screening of serum against arrays of candidate antigens and possesses considerable clinical potential for bioscreening in autoimmune diseases (Nezlin and Mozes, 1995).

#### 6.2.3 Cancer biomarkers detection

Dot blot assays have increasingly been explored for the detection of tumor associated biomarkers in blood or tissue extracts, presenting a simple and scalable alternative to conventional immunoassays. Such applications hold particular value in cancer screening, diagnosis, treatment monitoring, and prognostic evaluation. A notable example is human epidermal growth factor receptor 2 (HER2), a critical predictive and prognostic factor in breast cancer (Yu et al., 2023; Kumar et al., 2025). Tan and colleagues (Tan et al., 2011) utilized a dot blot assay to quantify serum HER2 levels in breast cancer patients. The results exhibited a strong correlation between serum HER2 concentrations and both tumor tissue HER2 status. These findings demonstrate that dot blot can be employed for HER2 monitoring, offering a cost-effective and efficient method that may complement existing assays in breast cancer management. Dot blot assays have also been applied in cholangiocarcinoma (CCA), a malignancy with high mortality, largely attributed to the lack of reliable non-invasive methods for early detection. Truong et al. (2021) identified cytokine-induced apoptosis inhibitor 1 (CIAPIN1) as a potential prognostic biomarker and employed dot blot to measure CIAPIN1 levels in serum from 159 CCA patients and 93 healthy controls. The analysis revealed significantly elevated CIAPIN1 levels in patients compared with controls. These findings indicate that dot blot detection of CIAPIN1 may facilitate the discrimination between malignant and non-malignant states and provide useful prognostic insights. Another clinically relevant application involves serological thymidine kinase 1 (STK1), an enzyme closely associated with cellular proliferation. Under physiological conditions, serum STK1 concentrations are maintained at low levels, whereas tumorigenesis typically leads to a notable elevation in its circulating levels. Chen et al. (2011) utilized a chemiluminescent dot blot assay to assess serum STK1 levels in a large cohort encompassing both healthy individuals and patients suspected of having cancer. This assay exhibited excellent diagnostic performance, with robust discriminative capacity between healthy subjects and suspected cancer cases, as reflected by favorable sensitivity and specificity profiles at the established clinical cutoff value. Importantly, this high-throughput approach enabled population-scale screening, distinguishing healthy individuals from cancer patients across common malignancies such as lung, breast, and colorectal cancers. Compared with ELISA, the dot blot method proved more cost-efficient and suitable for large-scale epidemiological studies or routine health examinations.

### 6.3 Food safety testing

#### 6.3.1 Food-grade protein residue detection

Food allergies constitute a substantial public health challenge, as residual protein contaminants in purified food-grade amino acid preparations may pose allergenic risks if not adequately controlled. Consequently, the detection and quantification of allergenic proteins are imperative for ensuring food purity and safety. A range of analytical methodologies have been employed for this purpose, and dot blot technology has emerged as a particularly valuable approach, characterized by its high sensitivity, operational simplicity, and minimal sample requirement (Belyi et al., 1995; Chaicumpa et al., 1995; Guillemin et al., 2009). When coupled with the fluorescent dye SYPRO Ruby, dot blot assays enable the detection of proteins across a broad molecular weight spectrum, including bovine serum albumin, lysozyme, ubiquitin, bovine insulin, and oxidized insulin B chain, with a detection limit as low as 0.1 ppm (Yamada et al., 2004). Utilizing bovine serum albumin as an internal standard, this method was applied to the analysis of 25 distinct food-grade amino acids and 2 food-grade nucleic acids. The results validated its efficacy in monitoring residual proteins in commercial preparations, thereby establishing it as a practical tool for mitigating allergen-related risks, safeguarding food quality, and ensuring consumer safety.

#### 6.3.2 Food allergen detection

Beyond routine quality control, dot blot has also demonstrated significant value in clinical allergy research, particularly in addressing the challenge of allergen cross-reactivity. Cashew allergy serves as a representative example, where patients frequently exhibit IgE-mediated responses not only to cashew proteins but also to other members of the Anacardiaceae family (e.g., pistachio, mango, and pink peppercorn) as well as to unrelated tree nuts (Brandstrom et al., 2016). Traditional diagnostic methods are generally limited to single-species testing, whereas dot blot enables simultaneous screening of multiple protein extracts immobilized on a single membrane. In a study of pediatric patients with suspected cashew allergy, dot blot analysis revealed that some individuals exhibited detectable IgE binding to cashew proteins (Bastiaan-Net et al., 2019). Among these sensitized children, 19% showed co-sensitization to other Anacardiaceae species, and 31% reacted to additional tree nuts. These results clearly delineate the sensitization spectrum of cashew-allergic patients and provide a foundation for further investigations into the mechanisms underlying cross-reactivity. Given the limited volume and precious nature of pediatric serum samples, dot blot offers a distinct advantage, as it requires only minute quantities of sample while still enabling robust analysis of cashew allergen cross-reactivity.

Dot blot has also been applied to evaluate allergens in novel food products. For instance, analysis of chickpea-based pasta revealed the presence of IgE-binding proteins corresponding to known chickpea allergens, including 7S globulin, 2S albumin, lipid transfer protein (LTP), and PR-10. Boiling the pasta released higher levels of allergenic proteins into the cooking water compared with boiling chickpea seeds, indicating that food processing influences allergen distribution while preserving their allergenic potential (Valdelvira et al., 2022). In summary, dot blot provides a rapid and high-throughput platform for defining the sensitization spectrum of allergic patients and identifying potential cross-reactive allergens. Beyond clinical research, this approach also supports broader applications in food safety and public health by contributing valuable data for allergen risk assessment.

## 7 Conclusion

The protein dot blot assay has established itself as a pivotal tool in life science research and medical diagnostics, owing to its simplicity, rapidity, high-throughput capability, and cost-effectiveness—attributes that make it particularly suited for large-scale screening and preliminary analyses. While current protocols offer adequate sensitivity and specificity for routine applications, challenges remain in detecting ultra-low-abundance proteins and analyzing complex biological matrices. Future advancements are poised to address these limitations through innovative labeling/detection systems and specificity-enhancing strategies. For sensitivity improvement, integration of quantum dot-labeled antibodies leverages exceptional photostability and high quantum yield to boost signal intensity and reduce background interference, facilitating low-abundance protein detection. Additionally, the utilization of nanomaterials such as gold nanoparticles and carbon nanotubes has demonstrated considerable potential for signal amplification. Through mechanisms including surface plasmon resonance, catalytic activity, or enhanced surface binding capacity, these materials can significantly amplify detection signals and potentially lower the detection limit to the femtogram scale or below, facilitating more precise measurement of trace proteins.

With regard to specificity, several strategies are being pursued. One is the custom-modified of antibodies to enhance binding affinity and reduce cross-reactivity, which directly decreases non-specific background. Another entails the adoption of sandwich-format dot blots, wherein one antibody immobilized on the membrane captures the target protein, and a second antibody recognizing a distinct epitope facilitates detection. This dual-recognition mechanism substantially reduces false positives, as a signal is generated only upon simultaneous binding of both antibodies. Moreover, advances in genetic engineering have enabled the production of single-domain antibodies, which are capable of binding unique epitopes inaccessible to conventional antibodies. When combined with dot blot assays, these engineered binders can markedly improve specificity in complex biological matrices such as serum or tissue homogenates. Collectively, these innovations hold the promise of enabling more reliable and sensitive protein detection in challenging experimental and diagnostic scenarios.

Protein dot blot also exhibits strong potential when integrated with other analytical approaches. Coupling dot blot with mass spectrometry offers a complementary workflow that leverages the strengths of both methods. Dot blot can first be applied to rapidly screen a large number of samples and identify those positive for the target protein. Only the positive samples are then subjected to mass spectrometry, which provides definitive identification and accurate quantification of the protein. This tiered approach not only improves screening efficiency but also conserves resources by focusing detailed analyses on relevant samples. Importantly, such a workflow opens new avenues for investigating protein-protein interactions and post-translational modifications, areas where precise characterization of protein identity and molecular changes is essential. In addition, combining dot blot with bioinformatics and large-scale data analysis will facilitate the construction of protein expression profiles, thereby providing valuable data to support disease mechanism research and biomarker discovery.

In clinical diagnostics, dot blot holds substantial promise for early disease screening and point-of-care testing. While dot blot assays for tumor biomarkers have been reported in research settings, their adoption in large-scale clinical practice remains limited. Future progress is likely to stem from the integration of dot blot with microfluidic platforms, which can automate key steps including sample preparation, loading, antibody incubation, and signal detection. Such integration would dramatically reduce time and increase throughput. For instance, a microfluidic dot blot device has the potential to analyze a drop of serum and simultaneously measure multiple cancer biomarkers within less than an hour. This type of miniaturized platform could provide clinicians with a rapid and effective tool for early cancer diagnosis, enabling multiplexed testing from minimal patient material. Beyond oncology, dot blot can also be adapted for the diagnosis of infectious diseases, particularly for multiplex serological screening. By spotting multiple pathogen-specific antigens on the same membrane, antibodies against several infectious agents can be detected from a single serum sample. For example, simultaneous detection of antibodies to hepatitis B virus, hepatitis C virus, and HIV has been demonstrated, offering improved efficiency and broader coverage in infection screening. Such an approach is especially valuable in primary medical institutions and during public health emergencies, where rapid and high-throughput testing of large populations is required. Together, these developments highlight the potential of dot blot to evolve from a research tool into a practical clinical platform for early detection and large-scale screening across diverse disease areas.

**FIGURE 2:**
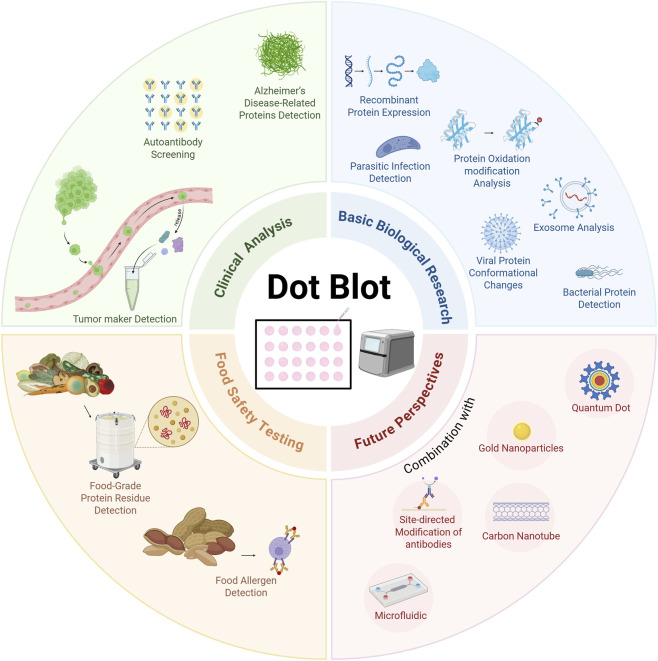
Applications and future perspectives of protein dot blot.

In summary, protein dot blot occupies a significant niche in the field of protein detection due to its unique advantages of simplicity, rapidity, and high throughput. With ongoing innovation in reagents, labeling chemistries, device integration, and data analysis, the technique is expected to achieve enhanced sensitivity and specificity, expand its range of applications, and play an increasingly important role in biomedical research, clinical diagnostics, food safety testing and public health surveillance (Figure 2).

Take-home message: Protein dot blot is a versatile, high-throughput immunoblotting technique enabling rapid qualitative/semi-quantitative protein analysis without electrophoresis, based on antigen-antibody specificity and solid-phase membrane immobilization. Boasting broad sample compatibility, minimal sample demand, simple operation, and picogram-level sensitivity, it excels in basic research, clinical biomarker screening (e.g., neurodegenerative diseases, cancer) and food safety testing. Despite limitations like narrow dynamic range, innovations such as nanomaterial signal amplification and integration with microfluidics will enhance its performance, solidifying its role as a pivotal tool linking research, diagnostics, and public health.
